# TIMP-1 Dependent Modulation of Metabolic Profiles Impacts Chemoresistance in NSCLC

**DOI:** 10.3390/cells11193036

**Published:** 2022-09-28

**Authors:** Wei Xiao, Pankaj Ahluwalia, Lan Wang, John Howard, Ravindra Kolhe, Amyn M. Rojiani, Mumtaz V. Rojiani

**Affiliations:** 1 Department of Pathology, Medical College of Georgia, Augusta University, Augusta, GA 30912, USA; 2 Department of Pathology, Penn State College of Medicine, Hershey, PA 17033, USA; 3 Room T3409, Penn State Cancer Institute, Penn State College of Medicine, Hershey, PA 17033, USA; 4 Department of Pharmacology, Penn State College of Medicine, Hershey, PA 17033, USA

**Keywords:** NSCLC, TIMP-1, chemoresistance, altered metabolism, bioinformatic analysis, CD44, STAT3

## Abstract

The development of chemoresistance remains a significant barrier to treating NSCLC. Alteration of cancer cell metabolism is an important mechanism for chemoresistance. This study explored the role of aberrant metabolism in TIMP-1-mediated chemoresistance. Bioinformatics analysis identified an association of high TIMP-1 with altered energy metabolism. We have defined the role of depolarized mitochondria through a reduction in lactate secretion, higher ROS levels in TIMP-1 KD cells and reduced GSH levels. TIMP-1 modulates the metabolic profile via acetylation of mitochondrial STAT3 and its interaction with CD44. Intriguingly, monomers of acetylated STAT3 were critical for altered metabolism, whereas STAT3 dimers abrogated this function. Further, the mitochondrial metabolic profile was also altered in a cisplatin-resistant clone of A549 cells. We also correlated the immunoexpression of CD44, STAT3 and TIMP-1 in patient samples. This study provided evidence that TIMP-1 alters the metabolic profile by modulating mitochondrial metabolism via the CD44-STAT3 axis through its effects on STAT3 acetylation. It also lent further support to the critical role of TIMP-1 in chemoresistance. Interrogation of the TCGA-LUAD dataset revealed perturbations in the critical modulator that can alter metabolic states in cancer cells. Higher expression of a five-gene signature, including *TIMP-1*, correlated with immunosuppressive cells and was found to be associated with overall survival. This study identified several metabolic mechanisms that could influence therapeutic options and prognosis in NSCLC patients.

## 1. Introduction

The largest number of cancer deaths worldwide is caused by lung cancer, with more than 85% of lung cancer classified as NSCLC. Adenocarcinomas remain the predominant histological phenotype of NSCLC. Platinum-based doublet therapy has been the standard for advanced-stage lung therapy; however, the development of chemoresistance remains an ongoing challenge.

Tissue inhibitor of metalloproteinase-1 (TIMP-1) is one of the four natural endogenous inhibitors of matrix-degrading enzymes and, therefore, ‘tumor suppressive’. However, it was shown to exhibit paradoxical tumor-promoting functions within the cancer tumor microenvironment [1].

Our recent study showed a role for TIMP-1 in chemoresistance in NSCLC [2]. Knocking down TIMP-1 in two NSCLC cell lines increased apoptosis upon treatment with Gemcitabine and Cisplatin. We showed that IL-6 expression closely correlated with TIMP-1 kinetics, and IL-6 was regulated by TIMP-1 exerting its effect via STAT3 activation. Knocking down TIMP-1 inhibited STAT3 phosphorylation.

Several mechanisms have been proposed for the development of drug resistance in cancer cells. These include cancer stem cells (CSCs), drug efflux, genetic instability, oncogenic signaling pathways, altered metabolism and transformed ECM [3,4,5]. The tumor microenvironment (TME), consisting of stromal cells, immune cells, ECM and vascular network, is an important determinant of tumor development. Within the TME, tumor heterogeneity allows cancer and the recruited host stromal cells to be in distinct phenotypic states. Metabolic pathways are additionally important in tumorigenic transformation, cancer progression and stemness maintenance of cancer stem cells [6].

Redox homeostasis is critical to the maintenance of cellular functions. However, cancer cells tilt the balance by causing a shift from oxidative phosphorylation to aerobic glycolysis. It is now becoming increasingly clear that cancer is a disease determined not only by the mutational accumulation of oncogenes and tumor suppressor genes but by altered mitochondrial metabolism. Mitochondria are the powerhouses of the cell, responsible for 90% of the ATP generated by the cell [7]. The mitochondria in the TME also have a dynamic nature and facilitate metabolic heterogeneity, playing a pivotal role in tumor progression, drug resistance and metastasis.

In this context, several studies have defined the role of mitochondria in the development and progression of lung cancer. GLUT1 was found to be of prognostic significance in NSCLC according to the histologic type [8]. Cruz-Bermudez et al. [9] found that in NCSLC, patient survival correlated with PGC-1α levels, a transcriptional coactivator important for mitochondrial biogenesis. It has been shown that EGFR signaling regulates metabolic pathways in lung adenocarcinoma [10]. Some of the NSCLC-specific mutations that alter the metabolic profile of these cancer cells cause an increase in lactate production within the TME [11]. Several studies have documented metabolic reprogramming in NSCLC with KRAS mutations [12].

We sought to comprehensively assess the role of aberrant metabolism due to TIMP-1 in the regulation of mitochondrial function to further delineate the mechanisms involved in TIMP-1 mediated chemoresistance. We explored molecular mechanisms, along with the verification of immune-expression patterns in surgical resection specimens from patients with lung adenocarcinoma. The study additionally performed a bioinformatics evaluation of subsets of patients with TIMP-1 dysregulation using the TCGA dataset.

## 2. Materials and Methods

### 2.1. Cell Culture shRNA Transduction

Human NSCLC cell lines (A549 and H460) were purchased from American Type Culture Collection (ATCC, Manassas, VA, USA). A549- and H460-derived cell clones, encoding non-target scrambled shRNA (-NT) or TIMP-1-specific knockdown shRNA (-KD) sequences, characterized in our previous studies [13] were used. A549 and its clones were cultured in an F-12K medium, and H460 and its clones were grown in RPMI Medium 1640 (Sigma-Aldrich, St. Louis, MO, USA) as per ATCC recommendations. Cells were cultured under normoxic (20% O_2_ with 5% CO_2_) or hypoxic conditions as indicated (1% O_2_ with 5% CO_2_). To generate knockdown clones of TIMP-1, we constructed TIMP-1 KD and NT clones with shRNA Lentiviral Particles (Sigma-Aldrich), as described in previous studies [2]. All human cell lines were authenticated using STR (or SNP) profiling within the last three years and were mycoplasma-free cells.

### 2.2. Reverse Transcription Quantitative Real-Time PCR (RT-qPCR)

RT-qPCR analysis was performed as previously described [2] to titrate the expression level of interested genes. Briefly, total RNA was extracted using RNAqueous™-4PCR Total RNA Isolation Kit (Invitrogen, Carlsbad, CA, USA), and 1 μg RNA was reverse-transcribed using an iScript cDNA synthesis kit (Bio-Rad, Hercules, CA, USA). Primer sequences are listed in Table S1. The amplification of cDNA was performed by real-time PCR on CFX Connect Real-Time PCR System (Bio-Rad) using iTaq Universal SYBR Green Supermix (Bio-Rad) with the following settings: 94 °C for 10 min, followed by 40 cycles of amplification at 94 °C for 15 s and 60 °C for 1 min, with an extension step of 30 s at 72 °C. Data analysis was performed based on the 2^−∆∆Ct^ method with normalization of the raw data to the housekeeping gene. Results were presented as the means ± SD. Gene expression was determined in triplicate in each reaction for at least three independent experiments.

### 2.3. Western Blotting

Whole-cell proteins, mitochondria-derived proteins, or nuclear-derived proteins were extracted using RIPA Lysis and Extraction Buffer (Thermo Fisher Scientific, Waltham, MA, USA) on ice supplemented with protease and phosphatase inhibitors (Thermo Scientific), combined with short sonication. Isolated mitochondria or nuclei were lysed in ice-cold RIPA buffer for 30 min with vortexing. Protein lysates were then separated on 4–20% SDS-PAGE (Bio-Rad) and transferred to PVDF membranes (MilliporeSigma, Burlington, MA, USA) and blocked in 5% non-fat milk (Bio-Rad) in Tris-buffered saline with 0.05% Tween-20 (TBS-T). The membranes were incubated overnight (4 °C) with primary antibodies against Ace-STAT3(K685), p-STAT3 (Y705), STAT3, CD44, Sirt5, Histone H3, beta-Tubulin, Porin and GAPDH as listed in Table S2. Horseradish peroxidase-conjugated secondary antibodies (SigmaAldrich 1:10,000) were applied to the membranes, and the protein expression levels were detected using the enhanced chemiluminescence system (SuperSignal^TM^ West Dural, Thermal Scientific, Waltham, MA, USA). Data were quantified by densitometry using ImageJ (version 1.5r) and normalized to reference genes as the loading control.

### 2.4. Mitochondria Isolation

The mitochondrial fraction was isolated using Mitochondria Isolation Kit for Cultured Cells (ab110171, Abcam, Boston, MA, USA) according to the manufacturer’s instructions. Briefly, NSCLC cells were washed with a pre-cooled PBS buffer three times and pelleted by centrifugation at 200× *g* for 5 min. The cells were frozen and thawed for three rounds to weaken cell membranes. Then the cells were suspended in Reagent A for incubation on ice for 10 min. Then the cellular suspension was homogenized in a pre-cooled Dounce homogenizer. The homogenates were centrifuged at 1000× *g* for 10 min at 4 °C and saved as supernatant #1. The pellet was resuspended in Reagent B, and the process was repeated to aid in cell rupturing. The homogenates were centrifuged and saved as supernatant #2. Supernatants #1 and #2 were combined and centrifuged at 12,000× *g* for 10 min at 4 °C. The pellet was suspended into Reagent C, supplemented with protease inhibitors (HaltTM Protease Inhibitor Cocktail, Thermo Fisher Scientific). The aliquots were frozen at −80 °C until use.

### 2.5. Nuclear and Cytoplasmic Protein Extraction

Nuclear and cytoplasmic proteins were extracted with a NE-PER™ Nuclear and Cytoplasmic Extraction Kit (Thermo Scientific™) following the manufacturer’s instructions. Briefly, the cell pellet was washed twice with cold PBS and centrifuged at 500 g for 3 min. Then cells were suspended in 200 μL of cytoplasmic extraction reagent I with vortexing. The suspension was incubated on ice for 10 min, followed by the addition of 11 μL of a second cytoplasmic extraction, reagent II, vortexed for 5 s, incubated on ice for 1 min and centrifuged for 5 min at 16,000× *g*. The supernatant fraction (cytoplasmic extract) was transferred to a pre-chilled tube. The insoluble pellet fraction, which contained crude nuclei, was resuspended in 100 μL of nuclear extraction reagent with vortexing for 15 s and incubated on ice for 10 min, then centrifuged for 10 min at 16,000× *g*. The resulting supernatant, constituting the nuclear extract, was used for the subsequent experiments.

### 2.6. Liquid Chromatography-Mass Spectrometry Analysis

Protein samples were separated with 10% SDS-PAGE and individually processed for liquid-chromatography mass spectrometry (LC-MS/MS) analysis as per the published method [14]. The raw MS and MS/MS spectrum for each sample was filtered and processed using the Proteome Discoverer software by Thermo Fisher Scientific (v1.4) and then submitted to the SequestHT search algorithm against the Uniprot human database (10 ppm precursor ion mass tolerance: 10 ppm, product ion mass tolerance: 0.6 Da, static carbamidomethylation of +57.021 Da). The percolator PSM validator algorithm was used for peptide spectrum matching validation and false discovery rate estimation. The specific sequences of STAT3-derived peptides used in alignment were LLQTAATAAQQGGQANHPTAAVVTEK, GLSIEQLTTLAEK, AILSTKPPGTFLLR and YLEQLHQLYSDSFPMELR.

### 2.7. Mitochondrial Membrane Potential Assay

A TMRE-Mitochondrial Membrane Potential Assay Kit (ab113852, Abcam) was used to quantify changes in mitochondrial membrane potential in live NSCLC cells, according to the manufacturer’s instructions. Briefly, carbonyl cyanide 4-(trifluoromethoxy) phenylhydrazone (FCCP, 5 μM) was added into active NSCLC cell suspensions, including appropriate control cell samples, and incubated for 10 min at 37 °C, 5% CO_2_. Tetramethylrhodamine ethyl ester (TMRE, 200 nM) was then added to the cell suspensions and incubated for another 30 min. Suspended cells were pelleted by 200× *g* for 5 min and washed with PBS buffer supplemented with 0.2% BSA. The fluorescence was detected by flow cytometry (LSRII) through the channel of 488 nm/575 nm (excitation/emission).

### 2.8. Glycolysis Cell-Based Assay

L-lactate, the product of glycolysis, produced by cultured cells and secreted into the medium, was quantified in proportion to the quantity of formazan produced by cell cultures using a Glycolysis Cell-Based Assay Kit (600450, Cayman Chemical, Ann Arbor, MI, USA) following the manufacturer’s instructions.

### 2.9. Reactive Oxygen Species (ROS) Assay

The cellular ROS level was used with the cell-permeant reagent 2′,7′–dichlorofluorescein diacetate (DCFDA) to quantitatively assess reactive oxygen species in live cell samples with a Cellular ROS Assay Kit (ab113851, abcam) following the manufacturer’s instructions. Briefly, grow adherent NSCLC cells in standard cell culture media, harvest cells and seed a dark, clear bottom 96-well microplate with 25,000 cells per well. Allow cells to adhere overnight, and the gently wash cells with included 1× buffer once. Incubate cells with the diluted DCFDA solution for 45 min at 37 °C in the dark. Remove DCFDA solution and measure fluorescence immediately with a flow cytometer (Biosciences, Franklin Lakes, NJ, USA). The mean fluorescence intensity was analyzed by FlowJo.V10 software (FlowJo, Ashland, OR, USA).

### 2.10. GSH/GSSG Assay

Total oxidized (GSSG) and reduced glutathione (GSH) was quantitatively determined with an EnzyChromTM GSH/GSSG Assay Kit (EGTT-100, BioAssay Systems, Hayward, CA, USA) following the manufacturer’s instructions. Briefly, cell pellet samples were homogenized in 200 µL of cold buffer (50 mM phosphate (pH 7.0), 1 mM EDTA and 20 µL of capturer). The homogenates were centrifuged at 10,000× *g* × 15 min at 4 °C, and the supernatants were transferred to clean tubes for deproteinization. All samples were deproteinized by preparing a solution of 5% metaphosphoric acid in distilled water. Absorbances were read at 412 nm at 0 min and 10 min.

### 2.11. Immunofluorescence (IF)

Cultured cells were seeded as a monolayer on a chamber polystyrene vessel tissue culture treated glass slide (BD Biosciences). After approximately 70% confluence was reached, cells were fixed in 4% paraformaldehyde for 10 min at room temperature, then washed 3 times with PBS. Cells were permeabilized using 0.2% Triton X-100 in PBS for 15 min and blocked with 5% goat serum in PBS with 0.1% Triton X-100 for 1 h. Cells were incubated with primary antibodies (1:200 diluted) in blocking buffer overnight at 4 °C. After washing three times with PBS-0.1% Triton X-100, fluorescence-conjugated secondary antibodies (1:500 diluted) were applied correspondingly. Isotype-specific IgG was used as the negative control. Nuclei were stained with 4′,6-diamidino-2-phenylindole (DAPI, Vectashield, Vector Laboratories, Burlingame, CA, USA). The analysis used a BZ-X Series fluorescence microscope (Keyence, Itasca, IL, USA).

### 2.12. Immunoprecipitation

Immunoprecipitation was performed as per the immunoprecipitation protocol utilizing magnetic separation from cell signaling technology (CST, Danvers, MA, USA) with minor modifications. Briefly, cell lysate was pre-cleared with protein G magnetic beads to reduce non-specific protein binding. Primary antibodies, STAT3 or CD44, were added to cell lysate at the appropriate dilution as recommended in the product datasheet. Cells were incubated, with rotation, overnight at 4 °C to form the immunocomplex. The immunocomplex solution was then transferred to the tube containing the pre-washed magnetic bead pellet for 20 min at room temperature with rotation. Beads were pelleted using a magnetic separation rack and washed at least three times. Appropriate isotypes IgG corresponding to primary antibodies were also applied to the immunoprecipitation. The precipitated protein on pellet beads was detected using western blotting.

### 2.13. Bioinformatics Evaluation

To identify the pathways associated with TIMP1 expression in the TCGA-LUAD dataset, we explored Wikipathways and the Panther database [15,16]. The LinkedOmics portal and LinkInterpreter module were used to evaluate the pathways associated with TIMP-1 expression [17]. The WikiPathways database features curated biological information of metabolic pathways that can be integrated with other features of other omics fields [18,19]. Network interactions were plotted using a molecular complex detection (MCODE) algorithm [20]. Cystoscape software was used to construct interacting genes and pathways [21]. The correlation between immune cell infiltration and gene expression was analyzed using a geneset co-expression analysis [22]. The PCA analysis, Kaplan Meier (KM) curves and log-rank distribution were analyzed for survival distribution using the GEPIA portal [23].

### 2.14. Statistical Analysis

Data represented the means ± SD for at least three independent experiments. The significance of differences between experimental variables was determined by Student’s *t*-test and one-way analysis of variance. Multiple comparisons of means were conducted using the least significant difference (LSD) test. The Mann-Whitney U-test was used to investigate the relationship between gene expression of two ethnicities. All statistical analyses were performed with GraphPad Prism 9 software and JMP Pro 16. In all figures statistical significance was denoted as follows: * *p* < 0.05; ** *p* < 0.01; *** *p* < 0.001; N/S—non-significant.

## 3. Results

### 3.1. Bioinformatics Assessment Associates Mitochondrial Gene Signature with TIMP-1

**Figure 1:**
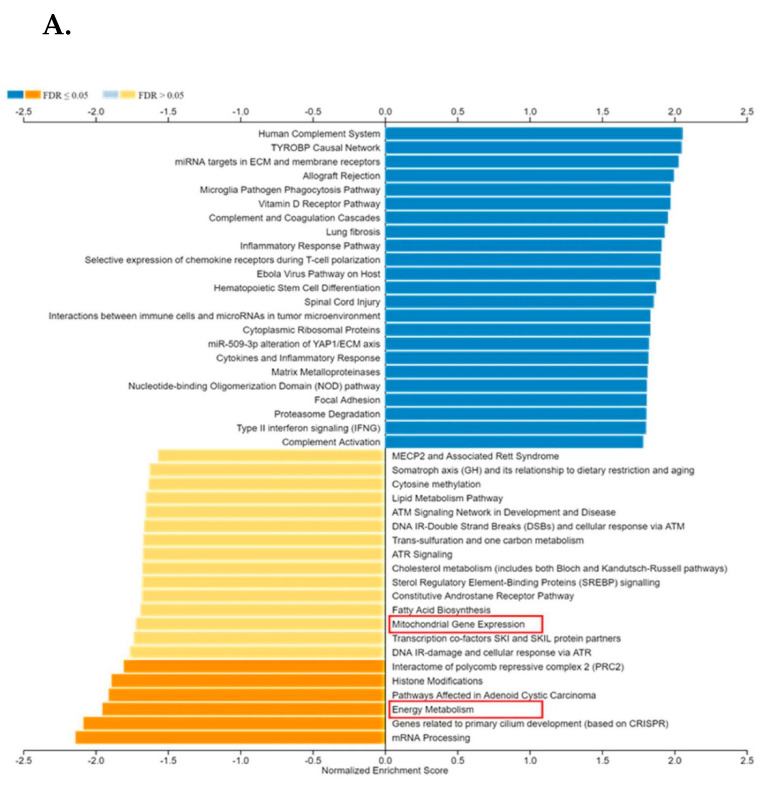

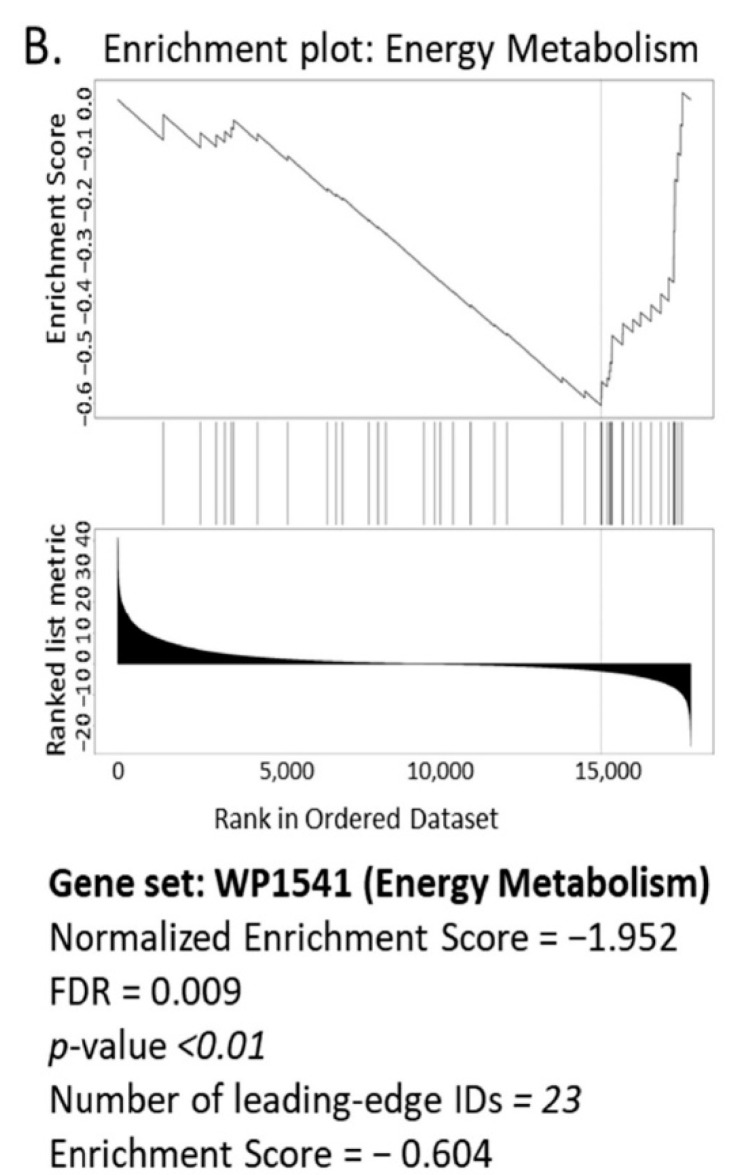
(**A**). Visualization of TIMP-1 enriched pathways through Wikipathways in TCGA lung cancer dataset. Red box: Further explored in manuscript. (**B**). GSEA plot of energy metabolism geneset.

The Wikipathways databases were explored to evaluate the TIMP-1-related biological networks. Among metabolic gene networks, mitochondrial gene expression and energy metabolism showed a negative enrichment score in relation to TIMP-1 expression in NSCLC patients. A geneset labeled WP1541 (energy metabolism) showed a significant normalized Enrichment score (NES) of −1.95 associated with TIMP expression (Figure 1A,B).

### 3.2. TIMP-1 Modulates Metabolic Profile in NSCLC

Upon identification of a perturbed energy metabolism correlating with high expression of TIMP-1, we proceeded to determine the metabolic features of NSCLC cells in relation to TIMP-1 modulation.

**Figure 2:**
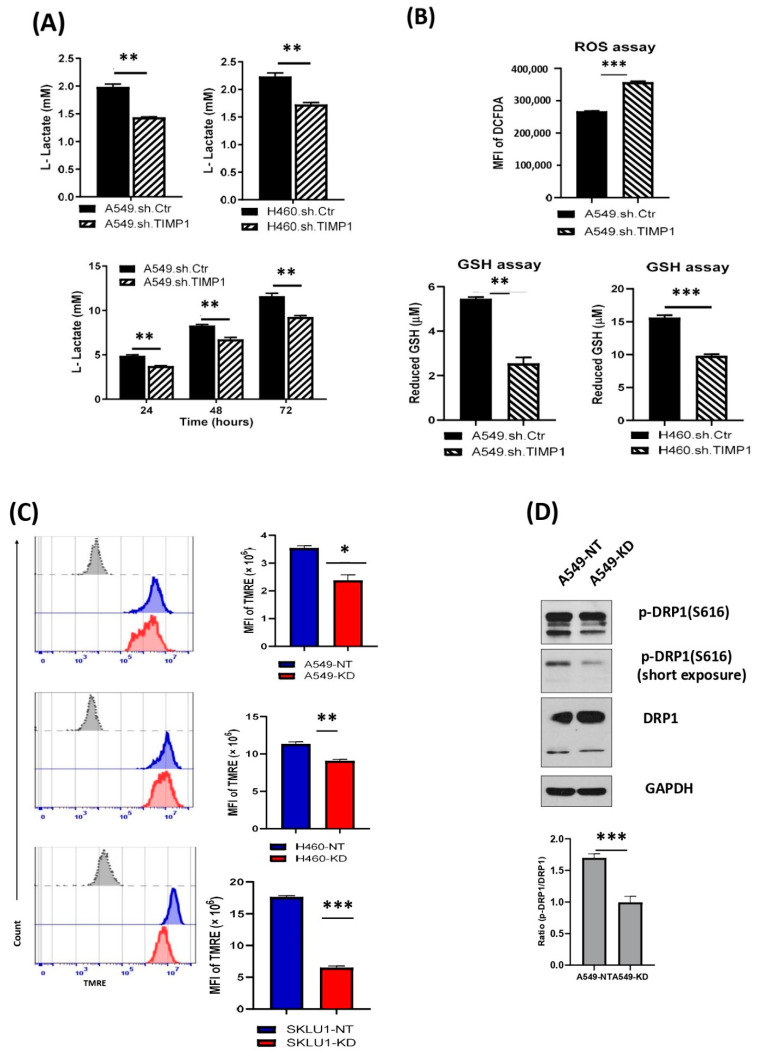
TIMP-1 modulates’ metabolic profiles in NSCLCs. (**A**) NSCLC cells (A549 and H460) derived TIMP-1 non-target (NT) control and knockdown (KD) clones were cultured in complete medium for three consecutive days. Supernatants were collected at 0, 24, 48 and 72 h. Equal volumes of supernatants were applied to measure L-lactate production at same indicated time points with glycolysis assay. Cell attachment time was defined as the 0 h of time point. (**B**) Logarithmically growing NSCLC cells (A549) derived TIMP-1 NT, and KD clones were incubated with DCFDA solution. The cellular ROS levels were measured by mean of the mean fluorescence intensity (MFI) of DCFDA by flow cytometry. Total reduced-glutathione (GSH) was quantitatively determined with NSCLC cells (A549 and H460) derived TIMP-1 NT and KD clones by GSH/GSSG assay. (**C**) The active mitochondrial membrane potential in live NSCLC cells (A549, H460 and SKLU1)-derived TIMP-1 NT and KD clones were labeled with TMRE (tetramethylrhodamine and ethyl ester) and measured by flow cytometry (grey, isotype control; blue, NSCLC cells derived non-target (NT) control; red, NSCLC cells derived TIMP-1 knockdown (KD) clones). (**D**) Mitochondrial fission-required DRP1 protein expression level was detected with NSCLC cells (A549) derived TIMP-1 NT and KD clones cell lysates by Western blot. Data were quantified by densitometry using ImageJ and normalized to reference genes as loading control. Data representative of three (**A**,**B**,**D**) and two (**C**) independent experiments. Statistical significances are indicated by * *p* < 0.05; ** *p* < 0.01; *** *p* < 0.001.

An important characteristic of the glycolytic phenotype is increased glucose metabolism and lactate secretion, even in the presence of oxygen. Utilizing NSCLC cell lines A549 and H460 and their TIMP-1 KD clones, we found reduced lactate secretion in TIMP-1 KD cells lines (Figure 2A), indicating that TIMP-1 expression correlated with aerobic glycolysis and lactate production.

Reactive oxygen species (ROS) production primarily occurs in the mitochondria because of metabolic reactions. Cancer cells undergo oxidative stress due to high demand for ATP fueling and aberrant proliferation resulting in increased ROS levels. At high levels, ROS can promote cellular damage and cell death, and cancer cells combat ROS by developing powerful antioxidant mechanisms, allowing them to curb ROS levels. We, therefore, sought to determine ROS levels in A549 TIMP-1 KD and NT clones. Figure 2B (left panel) shows that knocking down TIMP-1 increased ROS levels, making the cells more prone to cell damage and death.

The increase in ROS levels indicated the loss of the ROS-detoxifying scavenger pathways of increased antioxidants. Glutathione (GSH) was the most abundant antioxidant molecule in the cell and was crucial for redox homeostasis and cell survival. We found that reduced-GSH levels decreased in these cells compared with NT controls FIG2B (right panel).

Several cancers exhibit high mitochondrial membrane potential along with low levels of ROS [24]. Polarized mitochondria, with their high negative membrane potential, readily uptake the cationic dye TRME. Staining with TRME showed that TIMP-1 KD clones of NSCLC cell lines have a reduced intensity of this fluorescent probe, indicative of depolarized mitochondria (Figure 2C).

The altered membrane potential was confirmed by a Western blot of DRP, an important fission-specific protein activated by its phosphorylation at S616 (Figure 2C). We thus confirmed that knocking down TIMP-1 results in a reduction in aerobic glycolysis.

### 3.3. TIMP-1 Affects STAT3 Activation via Its Acetylation

Our earlier study showed that TIMP-1-induced IL-6 was functional via activation of the downstream STAT3 pathway, and phos-STAT3 was downregulated in the nuclear fraction along with decreased IL-6 levels upon knocking down TIMP-1 in both cell lines.

Having established that TIMP-1 modulates glycolysis, we sought to conduct an in-depth analysis of the signaling pathway mediated via the upregulation of IL-6 by TIMP-1. Recent years have seen emerging data indicating the role of STAT3 in cellular metabolism [25,26].

**Figure 3:**
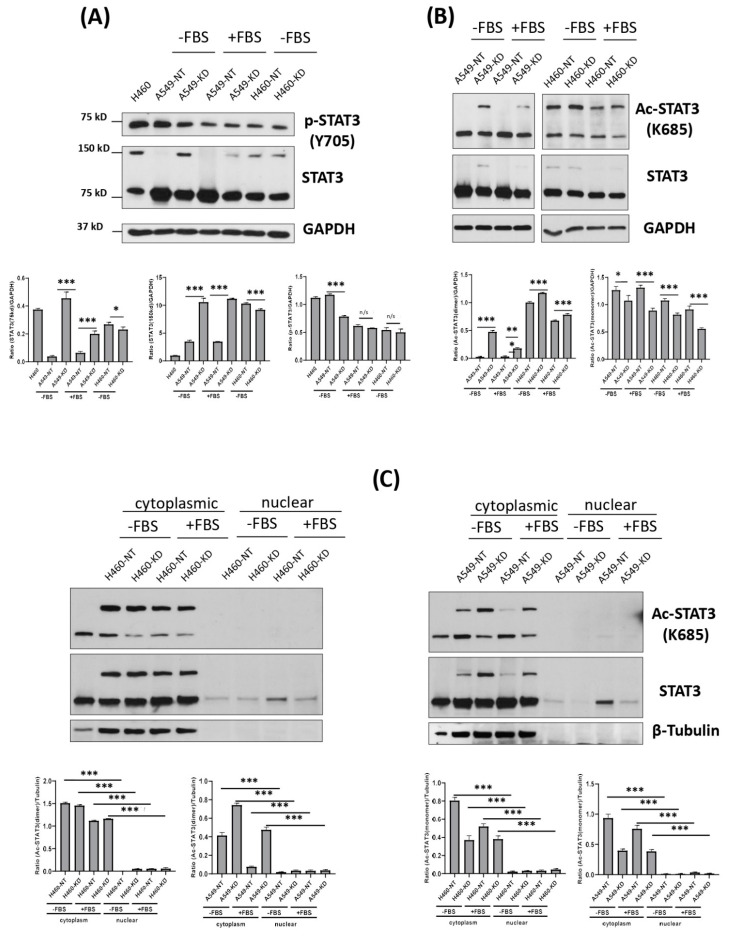

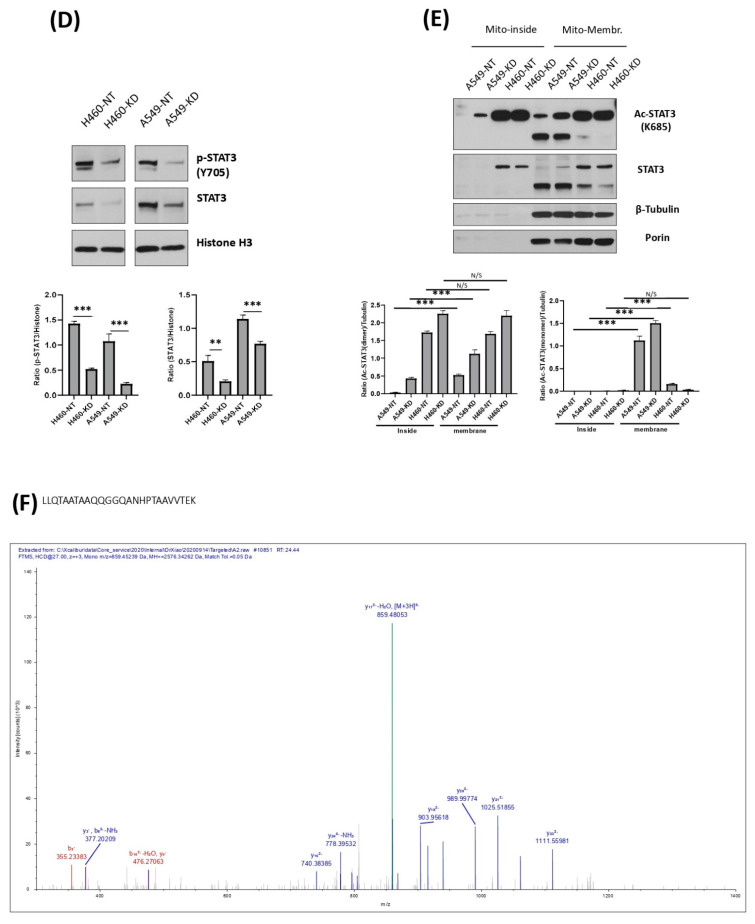

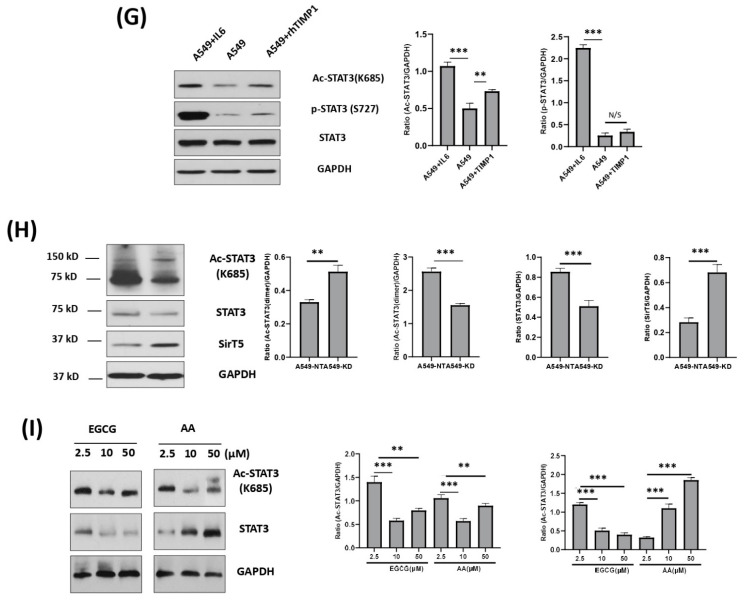
TIMP-1 affects STAT3 activation via its acetylation in NSCLCs. NSCLC cells (A549 and H460) derived TIMP-1 non-target (NT) control and knockdown (KD) clones were cultured in complete medium with or without 10% FBS for indicated hours. (**A**,**B**) Acetyl-STAT3, total STAT3 and GAPDH protein expression levels were detected with whole cellular lysates by Western blot. Data were quantified by densitometry using ImageJ and normalized to reference genes as loading control. (**C**) The nuclear and cytoplasmic protein was extracted and then detected for Acetyl-STAT3, total STAT3, β-actin and β-tubulin by Western blot. (**D**). Phosphor-STAT3, total STAT3 and histone H3 were detected with whole cellular lysates of NSCLC cells (A549 and H460)-derived TIMP-1 NT control and KD clones by Western blot. (**E**) The mitochondrial plasma and mitochondrial membrane protein were extracted with isolated mitochondria from NSCLC cells (A549 and H460)-derived TIMP-1 NT control and KD clones. Acetyl-STAT3, total STAT3, β-tubulin, porin, and histone H3 were detected by Western blot. (**F**) Representatives of mass spectrum of four specific peptides of STAT3. (**G**) NSCLC cells (A549) were treated with recombinant human IL-6 (100 ng/µL) and TIMP-1 (100 ng/µL) for 24 h. Whole-cell lysates were used to detect Acetyl-STAT3, phosphor-STAT3, and total STAT3 by Western blot. (**H**) Acetyl-STAT3, total STAT3 and Sirt5 were detected with NSCLC cells (A549) derived TIMP-1 NT control and KD clones by Western blot. (**I**) NSCLC cells (A549) were treated with EGCG and AA at indicated concentrations for 24 h. Cell pellets were detected for Acetyl-STAT3 and total STAT3. GAPDH was used as loading control. Data are representative of three independent experiments. Statistical significance is indicated by: N/S not significant, * *p* < 0.05; ** *p* < 0.01; and *** *p*< 0.001.

Upon reevaluating STAT3 phosphorylation status, we observed that in these cells, there was a presence of a high molecular weight band at approximately 150 kD, which was more pronounced in the TIMP-1 KD clones and appeared to be a dimer of STAT3 that could be visualized using an antibody to total STAT3 but not to pSTAT3 in our whole cell lysates, as seen in Figure 3A.

Since STAT3 is known to undergo other post-translational modifications, including acetylation on lysine residues, we determined that this putative dimer was acetylated STAT3 (Figure 3B).

To determine the localization of the acetylated STAT3, nuclear and cytoplasmic preps of cell lysate were prepared and probed with the total and acetylated form of STAT3 antibody following SDS-PAGE. We found that the acetylated form was exclusively present in the cytoplasm, and none was detected in the nucleus, as shown in Figure 3C.

On the other hand, phospho-STAT3 was readily observed in the nuclear prep and was downregulated in TIMP-1 KD clones (Figure 3D).

Since acetylated STAT3 has been shown to translocate to the mitochondria [27], we sought to determine if TIMP-1-modulated Acetyl-STAT3 had a function in the mitochondria. Figure 3E shows that both monomers and dimers of AcetylSTAT3 were present in the isolated mitochondria. Samples were subjected to mass spectrometry to confirm the identity of both the monomers and the dimers as bona fide STAT3 molecules. Figure 3F shows that the putative monomers and dimers were indeed STAT3 molecules. Furthermore, treatment of A549 cells with exogenous TIMP-1 or IL-6 for 24 h increased the level of Ac-STAT3 monomers compared with the control (Figure 3G). We also confirmed that the monomers and dimers were acetylated by showing that levels of SIRT5, a mitochondrial class III NAD-dependent deacetylase, increased as the levels of STAT3 monomer decreased in TIMP-1 KD clones. Additionally, the use of lysine acetyltransferase inhibitors (KATi) showed a decrease in Ac-STAT3 levels Figure 3H.

Our data indicated that the levels of the monomer form of acetyl-STAT3 decreased with TIMP-1 KD, whereas there was an increase in the level of acetyl-STAT3 dimers. It appeared that the dimer form was nonfunctional STAT3 as its levels increased in the less tumorigenic TIMP-1 KD cells. Protein dimerization has been shown as a means of function inhibition. For example, the inactivation of DAPK2 occurred by dimerization [28], and antiparallel dimers of STAT1 were required for its inactivation [29]. Hence, it was possible that dimerization of Acetyl-STAT3 monomers served to instantaneously abrogate its function.

Therefore, TIMP-1 modulation affected the levels of acetylated STAT3 dimers and monomers in the mitochondria, reinforcing a mitochondrial function in TIMP-1 mediated chemoresistance.

### 3.4. Correlation and Interaction of Mitochondrial STAT3 with CD44 upon TIMP-1 Modulation

Multiple studies have shown a significant correlation between CD44 and STAT3. Wu et al. [30] found that greater activation of STAT3, via increased IL-6 levels, allowed for a larger tumor burden with increased CD44 expression. A natural compound, ‘honokiol’, reduced secretion of IL-6 and phosphorylation of STAT3 with concomitant downregulated expression of ALDH and CD44 [31]. Furthermore, studies have shown the interaction of CD44 with STAT3 [32,33,34].

**Figure 4:**
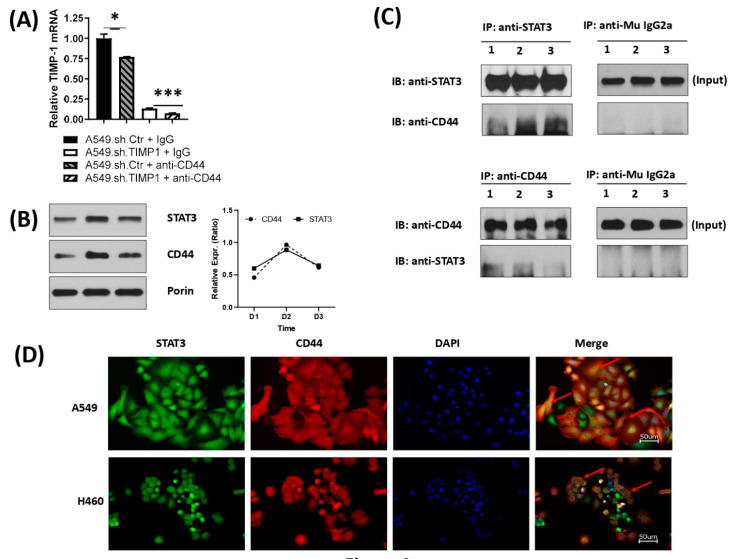
Correlation and interaction of mitochondrial STAT3 with CD44 upon TIMP-1 modulation. (**A**) NSCLC cells (A549) derived TIMP-1 non-target (NT) control and knockdown (KD) clones were treated with anti-CD44 antibody (Hermes-3, 15 µg/mL) or mouse-derived IgG2a k isotype for 24 h. Real-time quantitative RT-PCR was performed to measure the mRNA transcripts of TIMP-1 gene. (**B**) Mitochondria were isolated from NSCLC cells (A549) culture at indicated time points. The mitochondrial protein was extracted and then detected for CD44 and STAT3 by western blotting. (**C**) Whole lysates of NSCLC cells (A549) were incubated with anti-STAT3 or anti-CD44 antibodies separately at 4 °C overnight. Immunocomplex solutions were pulled down with protein-G magnetic beads. Precipitated immunocomplexes were detected for corresponding CD44 or STAT3 by western blotting. Whole-cell lysates were loaded as the input control. Data were quantified by densitometry using ImageJ and normalized to reference genes as loading control. (**D**) NSCLC cells (A549 and H460) were simultaneously stained with anti-STAT3 and anti-CD44 antibodies integrated with different fluorescence. The nuclei were stained with DAPI. Red arrows show the overlapping (yellow) areas of STAT3 and CD44. Data are representative of three independent experiments for (**A**,**B**), and two for (**C**,**D**). Each PCR was done in triplicate. Statistical significances are indicated by * *p* < 0.05 and *** *p* < 0.001.

Since TIMP-1 has been shown to induce CD44 expression [35,36], we interrogated whether TIMP-1 alteration of STAT3 had any effect on CD44. However, we first determined if ablation of CD44 levels using an antibody of CD44 affected TIMP levels. Figure 4A shows that treatment with antiCD44 antibodies caused a marked reduction in TIMP-1 mRNA levels in both the NT and KD clones of TIMP-1 compared with control IgG. We found that culturing parental A549 cells over the course of 72 h resulted in the accumulation of total STAT3 in the mitochondria. Interestingly, there was a concomitant mitochondrial accumulation of CD44, as shown in Figure 4B. As we found interdependent levels of the two proteins, we determined if there was any interaction between these proteins by carrying out an immunoprecipitation assay. Immunoprecipitation of CD44 co-immunoprecipitated STAT3 and vice versa (Figure 4C). Furthermore, immunocytochemistry showed colocalization of STAT3 with CD44 (Figure 4D). These correlative levels of CD44 and STAT3 in the mitochondria, along with co-immunoprecipitation, were strongly suggestive of mitochondrial function.

### 3.5. Mitochondrial Metabolic Profile Was Altered in Cisplatin-Resistant Clone of A549

Platinum drugs like cisplatin are known to generate extremely high ROS levels. Though moderate levels of ROS contribute to tumor formation and growth, high levels of ROS are detrimental to cancer cells causing cellular damage and death. One way by which cancer cells overcome this is by a concomitant increase in antioxidant levels to counteract ROS resulting in the development of chemoresistance. In our recent study, we selected a cisplatin-resistant clone of A549 (A549-Cis-R) cells by the repeated escalation of the drug dose over the course of 3 months. This clone was found to express higher TIMP-1 and IL-6 levels [2].

**Figure 5:**
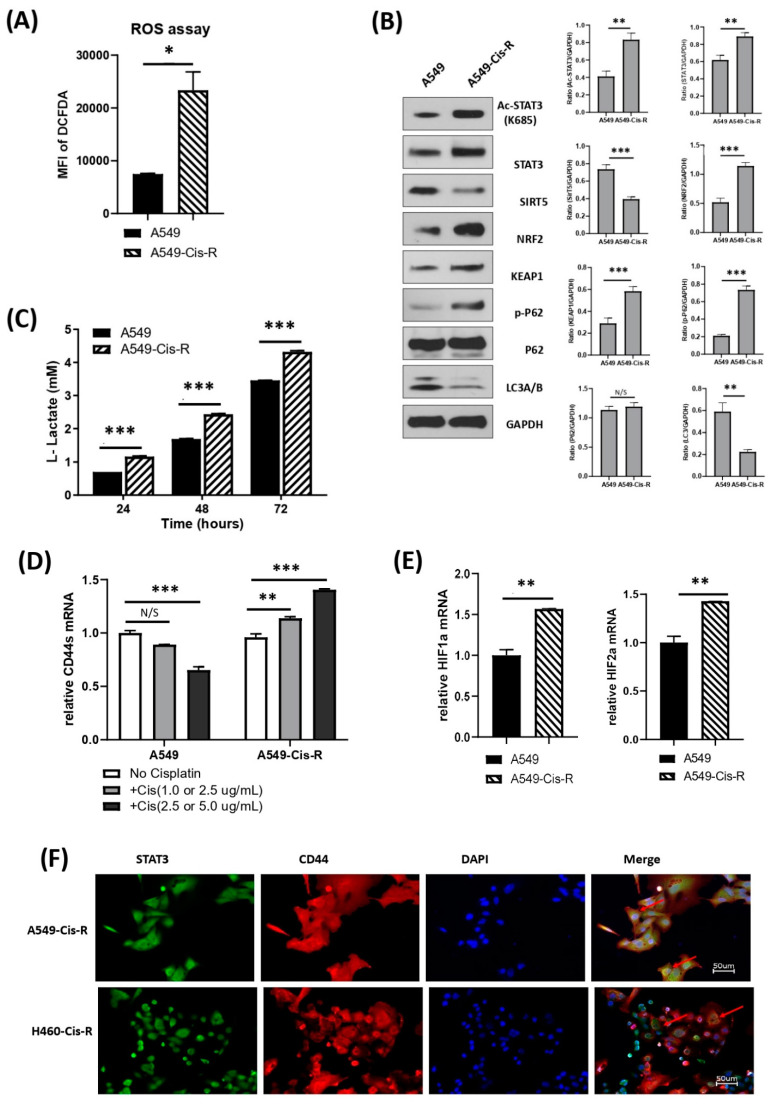
Changes in mitochondrial functions in the cisplatin-resistant clone of NSCLCs. (**A**) NSCLC cells (A549) and corresponding cisplatin-resistant cells (A549-Cis-R) were incubated with DCFDA solution. The cellular ROS levels were measured by mean of the mean fluorescence intensity (MFI) of DCFDA by flow cytometry. (**B**) The signaling molecules associated with mitochondrial functions were detected with whole lysates of A549 and A549-Cis-R. Data were quantified by densitometry using ImageJ and normalized to reference genes as loading control. (**C**) Equal volumes of supernatants from A549 cells and A549-Cis-R cells were applied to measure L-lactate production at same indicated time points with glycolysis assay. (**D**) A549 cells and A549-Cis-R cells were treated with indicated concentrations (A549: 1.0 µg/mL, 2.5 µg/mL; A549-Cis-R: 2.5 µg/mL, 5.0 µg/mL) for 24 h. Real-time quantitative RT-PCR was performed to measure the mRNA transcripts of CD44s gene. (**E**) Hif1 alpha and Hif2 alpha mRNA levels were determined by real-time quantitative PCR in A549 cells and A549-Cis-R cells. (**F**) Cisplatin-resistant cells (A549-Cis-R and H460-Cis-R) were stained simultaneously with anti-STAT3 and anti-CD44 antibodies integrated with different fluorescence. The nuclei were stained with DAPI. Data representative of 3 independent experiments for (**A**,**C**–**E**) or 2 for (**B**,**F**) with each PCR done in triplicate. Red arrows show the overlapping (yellow) areas of STAT3 and CD44. Statistical significances are indicated by: N/S not significant, * *p* < 0.05; ** *p* < 0.01; and *** *p* < 0.001.

We determined ROS levels in the A549-Cis-R clone. Figure 5A shows that compared to parental A549 cells, the resistant clone has significantly high ROS expression.

We then determined if there was a compensatory upregulation of antioxidant expression by evaluating the expression of NRf2, the transcription factor that serves as a master regulator of antioxidant gene expression. NRF2 function was negatively regulated by Keap1, which, under normal conditions, targets NRf2 for ubiquitin-dependent degradation. Indeed, NRf2 expression significantly increased in the A549-Cis-R clone compared with the parental A549, along with an increase in the expression of p62, which acted as a disrupter of Keap1/Nrf2 interaction. At the same time, the expression of other key proteins was detected. Acetyl-STAT3 monomer expression was higher in the Cis-R clone along with total STAT3, Nrf2 and p-P62. On the other hand, there was a decrease in SIRT5 expression, which was upregulated in TIMP-1 KD clones Figure 5B. Thus, the resistant clone was able to survive in the presence of very high ROS levels by increasing its antioxidant production. As these cells are known to more proficiently utilize glycolysis, we determined the lactate production profile. Figure 5C shows that lactate levels were comparatively higher in A549-Cis-R cells than in parental cells, indicating increased glycolysis.

To determine the status of the CD44/STAT3 axis in the cisplatin-resistant A549-Cis-R, we first determined the CD44 mRNA expression in these cells. Figure 5D shows that in the presence of increasing cisplatin concentration, CD44 mRNA levels decrease in parental A549. However, in the cisplatin-resistant clone A549-cis-R expressing high levels of TIMP-1, there was a constant increase in CD44 mRNA levels in the presence of an increasing concentration of cisplatin.

In our previous study, we showed that hypoxia increased TIMP-1 levels [2]. Others have made similar observations [37,38]. Several studies have also shown a correlation between TIMP-1 and HIF1α [39,40]. Multiple studies have shown cooperation between STAT3 and HIF1α [3,41,42]. Hypoxic environments significantly contribute to ROS generation as well as chemoresistance development, and HIF1α was a crucial player, as reviewed by Semenza [43]. We, therefore, determined HIF levels in the A549-Cis-R clone and found that there was a substantial increase in HIF1αand HIF2α mRNA levels compared with parental A549 cells (Figure 5E). Moreover, cisplatin-resistant clones of both the cell lines again showed colocalization of STAT3 and CD44, emphasizing the importance of these molecules in chemoresistance (Figure 5F).

### 3.6. CD44, STAT3 Immunohistochemistry of Archived Patient Samples of NSCLC

**Figure 6:**
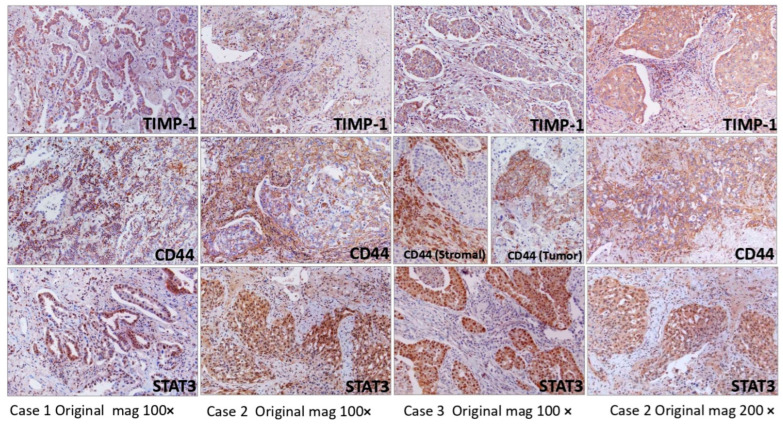
Formalin-fixed, paraffin-embedded surgical pathology specimens from three randomly selected patients with a diagnosis of NSCLC. Sections were immunoreacted with anti-TIMP-1, anti-CD44 and anti-STAT3. Original magnification 100× (first three columns) and 200× (last column).

Having established the important role of CD44 in TIMP-1-mediated chemoresistance, we determined the expression of CD44, STAT3 and TIMP-1 in archived NSCLC patient samples. As shown in Figure 6, there was a correlative expression of TIMP-1, CD44 and STAT3. Immunohistochemistry was performed on tumors from three randomly selected patients with a diagnosis of NSCLC. There was an excellent correlation between expression patterns of all three molecules (TIMP-1, CD44 and STAT3). TIMP-1 expression was cytoplasmic within tumor cells, whereas CD44 was seen as positive staining of cytoplasmic membranes of tumor cells and within multiple cell types in the stroma. STAT3 was strongly expressed in the nucleus with concomitant staining of the cytoplasm as well. NSCLC resections in the three patients immunoreacted with anti-TIMP-1, anti-CD44 and anti-STAT3.

### 3.7. Molecular Profiling of NSCLC Tumor Based on TIMP1 Expression

**Figure 7:**
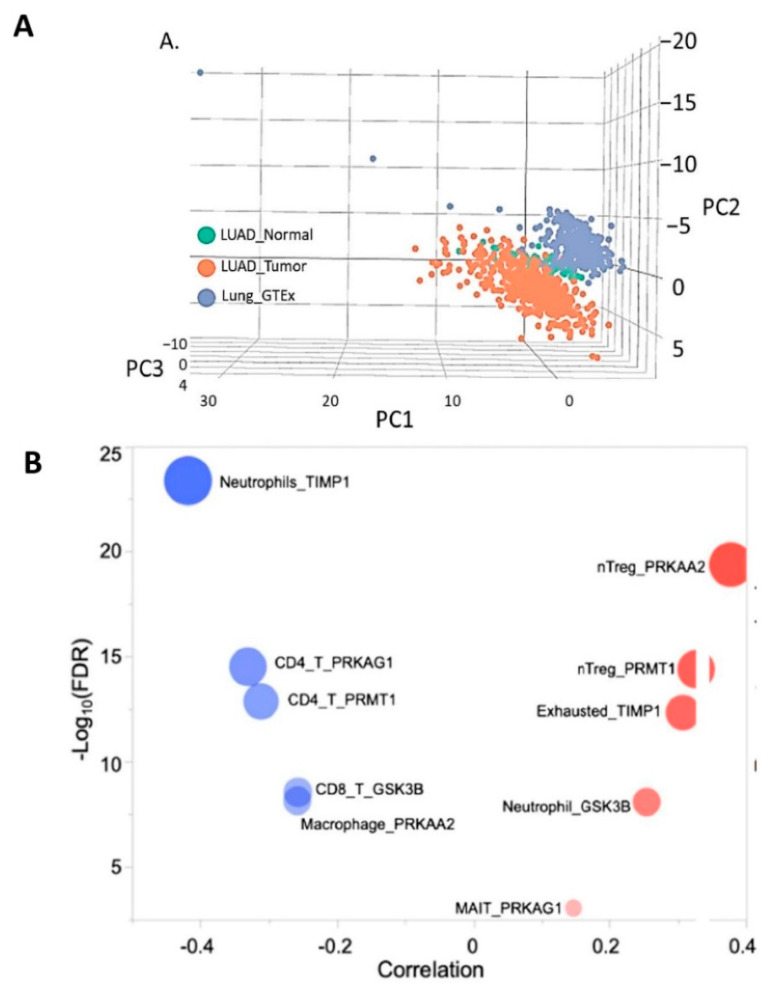

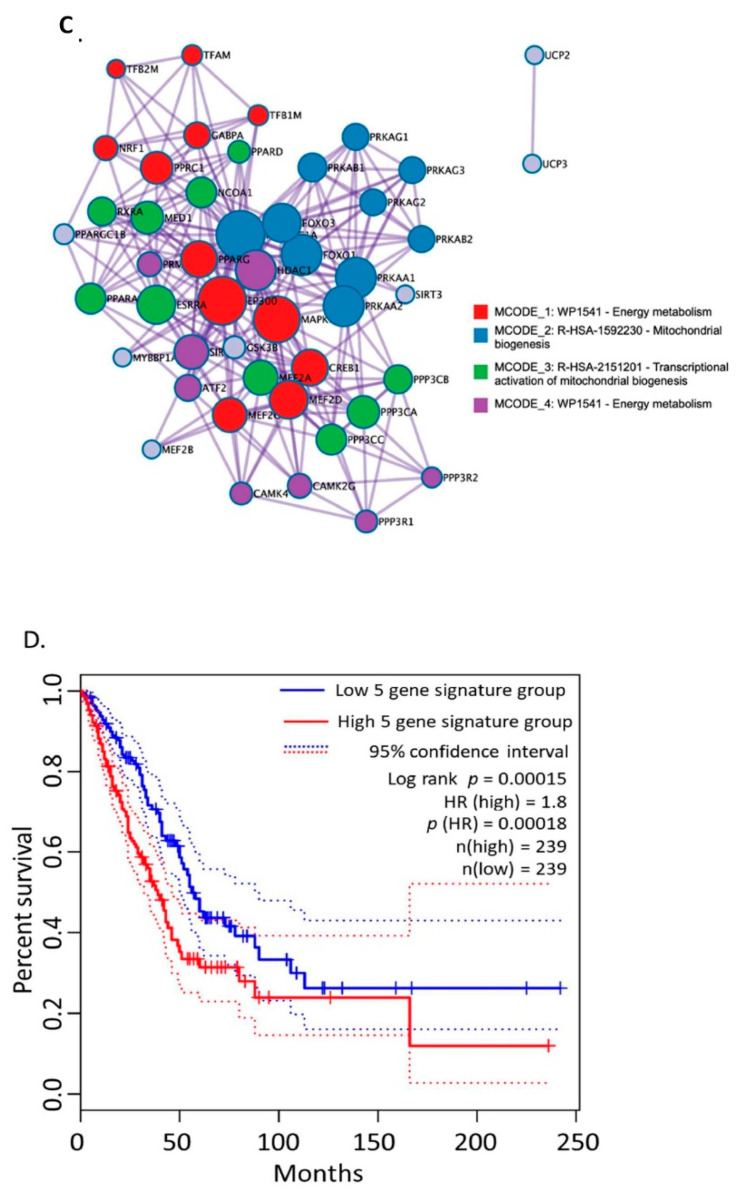
(**A**) Differential expression of WP-energy metabolism genes in LUAD tumor compared to control. (**B**) Network analysis showed distinct genes of energy metabolism, mitochondrial biogenesis and transcriptional activation of mitochondrial biogenesis. (**C**) Immune infiltration analysis showed a higher influx of immunosuppressive cells with the most perturbed genes. (**D**) A five-gene signature from energy metabolism panel showed prognostic significance in the lung cancer dataset.

To further assess the potential of TIMP1 expression in molecular profiling of NSCLC patients, we accessed the TCGA database. Enrichment analysis (energy metabolism) of 47 genes (Supplementary Table S3). Principal component analysis showed a distinct cluster of gene expression (Figure 7A). The MCODE algorithm showed three distinct clusters based on the association of individual genes. Immune cell infiltration was calculated for all genes and was ranked based on its association with gene expression. Immune infiltration showed an association of natural T-regulatory cells with higher expression of *PRKAA2* and *PRMT1*. Exhausted T cells, neutrophils and mucosal-associated invariant T (MAIT) cells were correlated with *TIMP1, GSK3B* and *PRKAG*1 genes (all correlations, Supplementary Table S4). On the other hand, immune cells with the better survival were CD4 T cells (*PRKAG1, PRMT1*), CD8 T cells (*GSK3B*), macrophage (*PRKAA2*) and neutrophils (*TIMP1*). The Kaplan-Meier curve of overall survival of the five-gene signature showed an association with worse survival.

## 4. Discussion

Mitochondria, as powerhouses of energy production in eukaryotes, are fundamental for several cellular pathways. These dynamic organelles are crucial for cell growth, signaling, cell cycle control and apoptosis. Mitochondrial dysfunction has been implicated in a myriad of human diseases, including diabetes and age-related neurodegenerative diseases [44,45]. However, its most notable role is in cancer metabolism [46]. Recent years have seen a plethora of publications on the role of metabolism in chemoresistance. Cancer cells exhibit the Warburg effect whereby cells utilize aerobic glycolysis in contrast to mitochondrial oxidative phosphorylation for the generation of ATP. This metabolic reprogramming is now recognized as a hallmark of tumors and is pivotal for cancer cell survival [47].

Myriad studies involving multiple cancers have shown that high TIMP-1 levels are associated with poor prognosis and reduced patient survival [1]. Although classically a tumor-inhibitory molecule, it acquires tumor-promoting functions within the TME, which are mainly MMP-independent. Our lab demonstrated its role in apoptosis, angiogenesis and, more recently, chemoresistance in NSCLC. The present study demonstrated the importance of increased glycolytic activity in the tumor-promoting and chemoresistance function of TIMP-1.

The determination of a gene expression profile is perhaps the most frequently employed approach used to dissect the molecular pathways involved in a wide range of genetic disorders, including cancers, since it provides the opportunity to evaluate possible transcriptome alterations at both gene and gene-network levels. To assess gene expression profiles associated with mitochondrial function in NSCLC and characterize the relationship between TIMP-1 and altered mitochondrial function, we compared NSCLC patient-derived tumor tissue and adjacent normal tissue targeting mitochondrial-related genes. Bioinformatics analysis indicated an association of high TIMP-1 levels with altered energy metabolism.

This led us to determine how aerobic glycolysis might be associated with TIMP-1. We found that knocking down TIMP-1 levels results in decreased lactate secretion, increased ROS levels with a concomitant decrease in antioxidant production and altered membrane potential. Together, these observations reinforce that TIMP-1 alters cellular metabolism. Therefore, high TIMP-1 levels known for poor prognosis and decreased patient survival mediates chemoresistance by altering oxidative phosphorylation in the mitochondria and promoting aerobic glycolysis.

In our previous study, we showed that TIMP-1 mediated chemoresistance via upregulation of IL-6. This IL-6 was functional via signaling through STAT3. Beyond its canonical function as a nuclear transcription factor, STAT3 exhibited non-canonical functions with a mitochondrial presence in phosphorylated and acetylated forms [27,48]. In recent years, the role of STAT3 in the glycolysis metabolic switch has gained considerable momentum [26,42,48]. This led us to interrogate STAT3′s role in TIMP-1 altered metabolism. We found that high TIMP-1 levels favored the accumulation of acetylated monomeric STAT3 exclusively in the mitochondria. Ablation of TIMP-1 levels resulted in increased dimerization of acetylated STAT3 monomers.

STAT3 dimers detected by conventional SDS-PAGE have been reported [49]. The formation of acetylated dimers of STAT3 (as confirmed by mass spectrometry) appeared to increase upon TIMP-1 knockdown. Since, at this point, the cells exhibited diminished features of aerobic glycolysis, these dimers may either be a nonfunctional form or may have a tumor suppressive role, one that allowed instantaneous abrogation of the monomer function. Dimer formation as a means for protein inactivation has been shown for STAT1 [29] and DAPK2 [28]. Further studies will be needed to dissect the function, if any, of these dimers.

After establishing a pivotal role of acetylated STAT3 in glycolysis, it was imperative to determine how STAT3 brings about the glycolytic response. In recent years, it has become increasingly clear that the CSC marker CD44 is an important player in redox regulation [50,51]. There is now evidence for its role in glucose metabolism [52,53,54]. A few studies have shown CD44 and STAT3 interaction affecting stemness and downstream signaling [32,33,34]. Dhar et al. [55] recently showed that STAT3 activates CD44. We, therefore, determined if CD44 levels were altered upon mitochondrial accumulation of STAT3 and found a concomitant increase in CD44 with STAT3. Thus, these proteins appeared to be co-regulated in the mitochondria, and their interaction was confirmed by the co-immunoprecipitation of both proteins and their colocalization in NSCLC cells. Although CD44 is a cell surface receptor, studies have shown its presence of CD44 in the nucleus and the mitochondria [56,57,58].

Furthermore, to eliminate any ambiguity in the role of TIMP-1 in aerobic glycolysis, we utilized a previously generated chemo-resistant clone of NSCLC cells [2]. Cisplatin-resistant A549 cells (A549-Cis-R), which expressed five-fold higher levels of TIMP-1 compared with parental A549 cells, exhibited increased lactate levels and very high levels of ROS with a compensatory increase in scavenger antioxidant pathways. Low to moderate levels of ROS allowed tumor growth; however, at high levels, ROS became detrimental to the tumor, and cancer cells combated high ROS by induction of a well-organized antioxidant defense system [59]. A549-Cis-R, fueled by high TIMP-1, displayed this classical response. Although SIRT5 has sometimes been shown to be tumor-promoting [60], several studies have shown a tumor suppressor function [61,62]. Alteration in the profile of relevant genes further reinforces the influence of mitochondrial metabolism in chemoresistance associated with TIMP-1 in NSCLC.

We also found that treating parental A549 cells with increasing cisplatin resulted in a decrease in CD44 mRNA levels. However, in A549-Cis-R, which has extremely high TIMP-1 levels, the CD44 mRNA level continued to increase with cisplatin treatment. It was possible that CD44s, which are usually upregulated during EMT and tumor progression (Brown et al., 2011), is expressed robustly in A549-Cis-R compared to the parental cells, which may have a pool of other isoforms such that lower CD44s responded to cisplatin treatment.

Accumulating evidence suggests that HIF1 α is a key player in the mitochondrial shift to aerobic glycolysis. The mitochondria are the largest contributor to cellular ROS, contributing almost 1% of the mitochondrial O_2_ consumption [63]. Hypoxic environments increase mitochondrial ROS allowing stabilization of HIF1α. HIF1α upregulates genes that promote cell survival, activate angiogenesis, and mediate a metabolic shift to glycolysis [64]. High mRNA levels of HIF1α in the A549-cisR clone, which has a 6-fold higher level of TIMP-1 mRNA, lend further support to the role of TIMP-1 in altered metabolism.

Immunohistochemistry was performed on randomly selected, archived surgical pathology patient samples with a diagnosis of NSCLC. All cases showed a positive correlation in the expression of TIMP-1, CD44 and STAT3. CD44 staining was seen as membranous staining in the tumor cells, and in some cases, there were many cells, likely inflammatory, within the stroma that were positive for CD44.

Recently, advances in genomics have provided novel tools to identify prognostic and predictive genes associated with several cancers. To further extend the translational relevance of our findings, we explored the TCGA lung cancer dataset. Top genes associated with overall survival showed a significant association with immunosuppressive immune cells. *PRKAA2* and *PRMT1* showed positive associations with T-regulatory cells. Immune cells such as T-reg cells have been associated with promoting tolerance and suppression of immune response. Increasing evidence has suggested its immunosuppressive mechanism to be a barrier to immune checkpoint inhibitors therapy [65,66]. Further, *TIMP1* and *PRKAG1* genes were found to be associated with exhausted T cells and mucosal-associated invariant T (MAIT) cells. Chronic exposure to antigens in the tumor microenvironment leads to a dysfunctional state of T cells [67]. Recently, a single cell analysis identified gene expression signatures associated with T cell exhaustion and anti-PD-1 efficacy in NSCLC patients [68]. The lower expression of several key genes showed an association with immune cells associated with a positive outcome in lung cancer. CD4 T cells were found to be associated with the expression of *PRKAG1* and *PRMT1*. Further, CD8 T cells were found to be associated with *GSK3B* expression.

## 5. Conclusions

Energy metabolism plays a key role in the initiation and progression of the tumor. TCGA analysis showed TIMP-1 is associated with energy metabolism in lung cancer. We set out to explore the interplay between energy metabolism and lung adenocarcinoma. TIMP-1 altered the metabolic profile by modulating mitochondrial metabolism via the CD44/STAT3 axis through its effects on STAT3 acetylation. A549 clones (cisplatin-resistant) showed altered ROS and antioxidant levels, further reinforcing that mitochondrial metabolism is a promising therapeutic target. This study also lends support to the critical role of TIMP-1 in chemoresistance and helps to explain why elevated TIMP-1 levels in serum are associated with poor prognosis. To understand the clinical relevance of energy imbalance and tumor microenvironment, we assessed the TCGA-LUAD dataset. The critical energy metabolism genes showed significant perturbation in tumor tissues compared to normal tissue. Further, higher expression of a five-gene signature showed a correlation with immunosuppressive cells and was found to be associated with overall survival. In summary, we identified a key role of energy metabolism in lung adenocarcinoma that could be clinically exploited based on its association with immune infiltration.

## Data Availability

Not applicable.

## Supplementary Materials

The following supporting information can be downloaded at: https://www.mdpi.com/article/10.3390/cells11193036/s1, Table S1: Primer Sequences; Table S2: Antibodies; Table S3: Gene list of ‘Energy Metabolism’ in WikiPathways; Table S4: Correlation of immune cells with the prognostic genes in LUAD dataset.
