# Supplementary material for: TIMP-1 Dependent Modulation of Metabolic Profiles Impacts Chemoresistance in NSCLC
Source: Cells. 2022 Sep 28;11(19):3036. doi: 10.3390/cells11193036 (PMC9562647; doi:10.3390/cells11193036)
Supplement: Supplementary file 1 [file cells-11-03036-s001.zip › cells-1884139-supplementary.pdf]

Supplementary Table S1: Primer Sequences

Gene	Forward	Reverse
TIMP-1	5′ATGGACTCTTGCACATCACTAC3′	5′GGGATGGATAAACAGGGAAACA3′
CD44s	5′CTGTACACCCCATCCCA GAC3′	5′TGTGTCTTGGTCTCTGGTAGC3′
β-Actin	5′CACTCTTCCAGCCTTCCTTC3′	5′GTACAGGTCTTTGCGGATGT3′

Supplementary Table S2: Antibodies

Antibody	Catalog number	Company	Dilution	Application
Ace-STAT3	2523	CST	1:500	WB
STAT3	9139	CST	1:1000/1:200	WB/IP
STAT3	12640	CST	1:1000	WB
CD44	3750	CST	1:1000/1:50	WB/IP
CD44	NBP2-29976	NOVUS	1:50	Neutralizing
Mouse IgG2a Isotype	61656	CST	1:50-1:200	IP
Sirt5	8779	CST	1:1000	WB
Histone H3	3638	CST	1:1000	WB
beta-Tubulin	86298	CST	1:1000	WB
Porin	4661	CST	1:1000	WB
GAPDH	SC-32233	Santa Cruz	1:2000	WB

Supplementary Table S3 : Gene list of 'Energy Metabolism' in WikiPathways.

Name	Database reference
ATF2	1386 (Entrez Gene)
CAMK2G	818 (Entrez Gene)
CAMK4	814 (Entrez Gene)
CREB1	1385 (Entrez Gene)

EP300	2033 (Entrez Gene)
ESRRA	2101 (Entrez Gene)
FOXO1	2308 (Entrez Gene)
FOXO3	2309 (Entrez Gene)
GABPA	2551 (Entrez Gene)
GSK3B	2932 (Entrez Gene)
HDAC1	3065 (Entrez Gene)
MAPK14	1432 (Entrez Gene)
MED1	5469 (Entrez Gene)
MEF2A	4205 (Entrez Gene)
MEF2B	100271849 (Entrez Gene)
MEF2C	4208 (Entrez Gene)
MEF2D	4209 (Entrez Gene)
MYBBP1A	10514 (Entrez Gene)
NCOA1	8648 (Entrez Gene)
NRF1	4899 (Entrez Gene)
PPARA	5465 (Entrez Gene)
PPARD	5467 (Entrez Gene)
PPARG	5468 (Entrez Gene)
PPARGC1A	10891 (Entrez Gene)
PPARGC1B	133522 (Entrez Gene)
PPP3CA	5530 (Entrez Gene)
PPP3CB	5532 (Entrez Gene)
PPP3CC	5533 (Entrez Gene)
PPP3R1	5534 (Entrez Gene)
PPP3R2	5535 (Entrez Gene)
PPRC1	23082 (Entrez Gene)
PRKAA1	5562 (Entrez Gene)
PRKAA2	5563 (Entrez Gene)
PRKAB1	5564 (Entrez Gene)
PRKAB2	5565 (Entrez Gene)
PRKAG1	5571 (Entrez Gene)
PRKAG2	51422 (Entrez Gene)
PRKAG3	53632 (Entrez Gene)
PRMT1	3276 (Entrez Gene)
RXRA	6256 (Entrez Gene)
SIRT1	23411 (Entrez Gene)
SIRT3	23410 (Entrez Gene)
TFAM	7019 (Entrez Gene)
TFB1M	51106 (Entrez Gene)
TFB2M	64216 (Entrez Gene)

UCP2	7351 (Entrez Gene)
UCP3	7352 (Entrez Gene)

Supplementary Table S4 : Correlation of immune cells with the prognostic genes in LUAD dataset

Cancer type	symbol	cell_type	cor	p_value	fdr	entrez
LUAD	TIMP1	Neutrophil	-0.4180165	9.1097E-26	4.4254E-24	7076
LUAD	PRKAG1	CD4_T	-0.3304533	3.8392E-16	3.1829E-15	5571
LUAD	PRMT1	CD4_T	-0.3112584	2.0946E-14	1.4183E-13	3276
LUAD	PRKAA2	Infiltration Score	-0.2814733	5.9739E-12	4.0369E-11	5563
LUAD	PRMT1	NKT	-0.2710017	3.7388E-11	1.6045E-10	3276
LUAD	GSK3B	CD8_T	-0.2579599	3.2943E-10	7.5107E-09	2932
LUAD	PRKAA2	Macrophage	-0.2570321	3.8287E-10	2.9085E-09	5563
LUAD	PRKAA2	NKT	-0.2468433	1.9188E-09	6.9806E-09	5563
LUAD	PRKAA2	Gamma_delta	-0.2333536	1.4554E-08	1.6062E-07	5563
LUAD	PRKAA2	DC	-0.2054671	6.57E-07	6.1509E-06	5563
LUAD	PRKAG1	NKT	-0.199614	1.3721E-06	3.6804E-06	5571
LUAD	TIMP1	Gamma_delta	-0.185607	7.3287E-06	3.8765E-05	7076
LUAD	TIMP1	Th17	-0.1769486	1.9434E-05	8.5507E-05	7076
LUAD	PRKAG1	NK	-0.1673574	5.4288E-05	0.00015432	5571
LUAD	PRKAA2	MAIT	-0.1642074	7.5163E-05	0.00017821	5563
LUAD	TIMP1	CD4_naive	-0.1601724	0.00011304	0.00308991	7076
LUAD	PRKAA2	Tfh	-0.1511535	0.00027185	0.00065761	5563

LUAD	PRMT1	Th17	-0.1483219	0.00035458	0.00118467	3276
LUAD	GSK3B	Tfh	-0.1462394	0.00042981	0.00100306	2932
LUAD	PRMT1	Infiltration Score	-0.1446071	0.0004989	0.00113281	3276
LUAD	PRKAG1	Cytotoxic	-0.1425506	0.00060065	0.00221562	5571
LUAD	PRKAA2	Th2	-0.1423377	0.00061222	0.00170171	5563
LUAD	PRKAG1	Infiltration Score	-0.142323	0.00061302	0.00136839	5571
LUAD	PRKAG1	Tfh	-0.1422545	0.00061679	0.00140255	5571
LUAD	GSK3B	Exhausted	-0.1369727	0.00098159	0.00223184	2932
LUAD	PRMT1	Th2	-0.1361013	0.00105818	0.00279517	3276
LUAD	GSK3B	DC	-0.1321506	0.0014795	0.00522866	2932
LUAD	PRMT1	Tfh	-0.1307136	0.00166763	0.00351779	3276
LUAD	TIMP1	NKT	-0.130271	0.00172987	0.00319344	7076
LUAD	PRKAA2	CD4_T	-0.1256523	0.00251869	0.00455248	5563
LUAD	PRMT1	Macrophage	-0.1248106	0.00269371	0.00607643	3276
LUAD	PRMT1	MAIT	-0.1224088	0.00325573	0.00597017	3276
LUAD	TIMP1	MAIT	-0.1019155	0.01440374	0.0237065	7076
LUAD	PRKAA2	NK	-0.0980819	0.0185461	0.0326481	5563
LUAD	GSK3B	Effector_memory	-0.0915155	0.02807687	0.0463474	2932
LUAD	GSK3B	CD4_T	-0.0863781	0.03822388	0.05661762	2932
LUAD	PRKAG1	Gamma_delta	-0.0818863	0.0494951	0.08886882	5571
LUAD	PRKAA2	Cytotoxic	-0.079163	0.05759541	0.11205747	5563

LUAD	GSK3B	Infiltration Score	-0.0763086	0.0672354 2	0.10078 782	2932
LUAD	PRKAG1	Tr1	-0.0745838	0.0736768	0.14185 965	5571
LUAD	PRMT1	NK	-0.0741133	0.0755187 9	0.11362 081	3276
LUAD	TIMP1	Effector_ memory	-0.0697378	0.0945031 1	0.13779 186	7076
LUAD	GSK3B	MAIT	-0.0694464	0.0958919 7	0.13351 937	2932
LUAD	PRKAG1	Th2	-0.0669887	0.1082648 9	0.16681 656	5571
LUAD	TIMP1	Th2	-0.0647122	0.1208188 4	0.18312 219	7076
LUAD	GSK3B	Gamma_d elta	-0.0613378	0.1414821 8	0.21470 062	2932
LUAD	TIMP1	NK	-0.0586135	0.1600580 6	0.22074 963	7076
LUAD	PRMT1	Neutrophil	-0.0503059	0.2280170 7	0.36215 22	3276
LUAD	PRKAG1	CD8_naive	-0.0440335	0.2914164 1	0.39926	5571
LUAD	GSK3B	NK	-0.0432495	0.3001009 4	0.37857 93	2932
LUAD	PRKAG1	iTreg	-0.0409753	0.3262564 8	0.42175 797	5571
LUAD	PRMT1	DC	-0.0384951	0.3564151	0.48156 483	3276
LUAD	PRMT1	CD8_naive	-0.0357795	0.3913799 6	0.50254 439	3276
LUAD	PRMT1	CD4_naive	-0.0349522	0.4024322 2	0.64929 615	3276
LUAD	PRKAG1	Monocyte	-0.0327477	0.4327779	0.57699 629	5571
LUAD	GSK3B	Th1	-0.0308376	0.4601073	0.56184 618	2932
LUAD	GSK3B	Th17	-0.0238089	0.5685058 5	0.66639 72	2932
LUAD	PRMT1	Tr1	-0.0230136	0.5814970 4	0.69094 315	3276
LUAD	PRKAG1	Bcell	-0.0229343	0.5827991 8	0.68091 791	5571

LUAD	PRKAG1	Macrophage	-0.0179864	0.66663461	0.73285974	5571
LUAD	GSK3B	Cytotoxic	-0.0176333	0.67279592	0.76573092	2932
LUAD	PRMT1	Central_memory	-0.0131528	0.75276506	0.83109869	3276
LUAD	PRKAG1	Central_memory	-0.0107635	0.79658414	0.86234649	5571
LUAD	PRKAA2	Tr1	-0.0101921	0.80716379	0.86915667	5563
LUAD	GSK3B	Bcell	-0.0024282	0.9536291	0.96888948	2932
LUAD	GSK3B	Th2	-0.0017864	0.96587652	0.97574927	2932
LUAD	GSK3B	Tr1	-0.00175	0.96657054	0.97876671	2932
LUAD	PRKAG1	CD4_naive	0.00196561	0.9624556	0.9830345	5571
LUAD	TIMP1	CD8_naive	0.00275445	0.94740704	0.9638369	7076
LUAD	GSK3B	CD4_naive	0.00511021	0.90259859	0.9553593	2932
LUAD	PRKAA2	CD4_naive	0.00676073	0.87137915	0.94017101	5563
LUAD	PRMT1	Gamma_delta	0.00818558	0.84458625	0.88819237	3276
LUAD	GSK3B	nTreg	0.00904565	0.82849793	0.85588559	2932
LUAD	GSK3B	CD8_naive	0.0103217	0.80476169	0.86206278	2932
LUAD	PRMT1	iTreg	0.01909397	0.64745431	0.7249546	3276
LUAD	GSK3B	Macrophage	0.02199437	0.59834511	0.67339418	2932
LUAD	TIMP1	Tfh	0.02377734	0.56901891	0.64110891	7076
LUAD	PRKAA2	Neutrophil	0.0251996	0.54612796	0.67786224	5563
LUAD	PRKAA2	CD8_T	0.027304	0.51311544	0.63407354	5563
LUAD	TIMP1	Macrophage	0.02903601	0.4867438	0.57131814	7076

LUAD	PRKAG1	Neutrophil	0.03154835	0.4498268	0.59001537	5571
LUAD	PRMT1	Cytotoxic	0.03405167	0.41467093	0.53646782	3276
LUAD	PRKAG1	Exhausted	0.03531045	0.39762322	0.48077591	5571
LUAD	PRKAG1	Th1	0.04111601	0.32459602	0.4245352	5571
LUAD	GSK3B	Central_memory	0.05118401	0.21999109	0.33746321	2932
LUAD	PRMT1	Monocyte	0.05523213	0.18560198	0.31119275	3276
LUAD	PRMT1	Bcell	0.05760266	0.16739973	0.25684773	3276
LUAD	PRKAA2	Exhausted	0.05877107	0.15893589	0.21980635	5563
LUAD	PRKAA2	Bcell	0.05912999	0.15640186	0.24323546	5563
LUAD	TIMP1	Monocyte	0.06609578	0.11306045	0.21336955	7076
LUAD	GSK3B	Monocyte	0.06777747	0.10416367	0.19994602	2932
LUAD	PRKAA2	Monocyte	0.08083857	0.05249093	0.1179312	5563
LUAD	PRKAA2	CD8_naive	0.08989576	0.0309916	0.06390385	5563
LUAD	PRKAG1	Th17	0.09348256	0.02485668	0.05054218	5571
LUAD	TIMP1	Infiltration Score	0.09820843	0.0183943	0.03118777	7076
LUAD	PRKAG1	CD8_T	0.09990123	0.0164658	0.04149126	5571
LUAD	PRKAA2	Th17	0.10428148	0.01227462	0.02749115	5563
LUAD	GSK3B	iTreg	0.10888856	0.00891154	0.01920639	2932
LUAD	PRKAA2	iTreg	0.11211565	0.00707216	0.01564375	5563
LUAD	PRKAG1	DC	0.11213746	0.00706098	0.01980214	5571
LUAD	GSK3B	NKT	0.11362499	0.00633493	0.01074368	2932

LUAD	TIMP1	CD4_T	0.11610871	0.0052708<br>5	0.00905<br>54	7076
LUAD	TIMP1	Bcell	0.12065458	0.0037313<br>8	0.01087<br>647	7076
LUAD	PRKAA2	Effector_<br>memory	0.13359093	0.0013106<br>7	0.00281<br>813	5563
LUAD	TIMP1	Cytotoxic	0.1351836	0.0011447<br>5	0.00390<br>798	7076
LUAD	PRKAA2	Th1	0.13538504	0.0011252<br>1	0.00310<br>097	5563
LUAD	PRKAA2	Central_m<br>emory	0.14479983	0.0004902<br>4	0.00205<br>293	5563
LUAD	PRKAG1	MAIT	0.14618683	0.0004318<br>9	0.00090<br>943	5571
LUAD	TIMP1	Th1	0.15846648	0.0001339<br>4	0.00045<br>625	7076
LUAD	TIMP1	DC	0.19004145	4.369E-06	3.2953E<br>-05	7076
LUAD	PRMT1	Th1	0.21065576	3.3587E-07	1.9248E<br>-06	3276
LUAD	TIMP1	Central_m<br>emory	0.22831539	3.0072E-08	4.3003E<br>-07	7076
LUAD	PRMT1	Effector_<br>memory	0.23487673	1.1649E-08	5.1534E<br>-08	3276
LUAD	TIMP1	iTreg	0.24706407	1.8544E-09	1.6455E<br>-08	7076
LUAD	TIMP1	nTreg	0.24989702	1.1925E-09	3.71E-<br>09	7076
LUAD	PRMT1	CD8_T	0.25343964	6.8135E-10	1.4683E<br>-08	3276
LUAD	GSK3B	Neutrophil	0.25459947	5.6621E-10	8.4522E<br>-09	2932
LUAD	TIMP1	Tr1	0.25481692	5.4683E-10	1.3511E<br>-08	7076
LUAD	PRKAG1	Effector_<br>memory	0.26127232	1.917E-10	1.0803E<br>-09	5571
LUAD	TIMP1	CD8_T	0.26247244	1.5725E-10	3.8104E<br>-09	7076
LUAD	PRKAG1	nTreg	0.27526551	1.7886E-11	6.4751E<br>-11	5571
LUAD	PRMT1	Exhausted	0.27997189	7.8081E-12	6.2561E<br>-11	3276

LUAD	TIMP1	Exhausted	0.30771147	4.2516E-14	4.7948E -13	7076
LUAD	PRMT1	nTreg	0.32694882	8.141E-16	4.2085E -15	3276
LUAD	PRKAA2	nTreg	0.37781664	5.535E-21	4.3893E -20	5563
